# Effect of planned pauses versus continuous energy restriction on weight loss and attrition: a systematic review

**DOI:** 10.1002/oby.23976

**Published:** 2024-01-21

**Authors:** Gina M. Wren, Dimitrios A. Koutoukidis, Jadine Scragg, Elena Tsompanaki, Alice Hobson, Susan A. Jebb

**Affiliations:** ^1^ Nuffield Department of Primary Care Health Sciences University of Oxford, Radcliffe Observatory Quarter Oxford UK; ^2^ National Institute for Health Research Oxford Biomedical Research Centre Oxford University Hospitals NHS Foundation Trust Oxford UK

## Abstract

**Objective:**

The objective of this study was to investigate whether pausing a weight loss program for a defined period of time could enhance weight loss and reduce attrition.

**Methods:**

Five databases and two trial registries were searched from inception to July 2023. Randomized‐controlled trials of adults with overweight and/or obesity were included if they compared planned‐pause interventions with continuous energy restriction (CER), usual care, or a minimal intervention. To be included, the weight loss intervention must have incorporated a pause of at least 1 week. Pooled mean differences for weight change and risk ratios for attrition were calculated using random‐effects meta‐analyses.

**Results:**

Nine intervention arms (*N* = 796 participants, 77% female) were included. Pooled results did not detect a significant difference in weight change between planned pauses and CER interventions at the end of the active intervention at a median 26 weeks (planned pauses vs. CER mean: −7.09 vs. −7.0 kg; mean difference: −0.09 kg; 95% CI: −1.10 to 0.93) or at final follow‐up at a median 52 weeks (planned pauses vs. CER mean: −6.91 vs. −6.19 kg; mean difference: −0.72 kg; 95% CI: −2.92 to 1.48). There was no difference in attrition between planned pauses and CER interventions at the end of the active intervention (risk ratio: 1.20, 95% CI: 0.82 to 1.75) or at final follow‐up (risk ratio: 1.04, 95% CI: 0.89 to 1.22).

**Conclusions:**

Planned pauses were consistently found to be no more or less effective than CER for weight loss or attrition.

**Study Importance**

**What is already known?**

- Intermittent energy restriction has been proposed to increase dietary adherence due to its more flexible nature, which could lead to improved weight loss outcomes.
- Previous reviews have considered short periods of intermittent energy restriction (e.g., the 5:2 diet), but the effect of pausing the period of energy restriction for longer (at least a week) remains unclear.

**What does this review add?**

- We examined nine intervention arms with 796 participants that implemented a “planned pause” of at least 1 week in a weight loss program compared with continuous energy restriction (CER).
- There was no evidence that planned pauses reduced attrition compared with CER.
- For people living with overweight or obesity, planned pauses were found to be no more or less effective than CER for weight loss.

**How might these results change the direction of research or the focus of clinical practice?**

- The findings provide consistent evidence that planned pauses could be incorporated into a weight loss intervention without adverse consequences.
- Longer pauses (≥2 weeks) might be more effective than shorter pauses (1 week).
- Advice on maintaining weight stability during the planned pause rather than returning to the previous diet might be beneficial.

## INTRODUCTION

The conventional approach to weight loss involves continuous energy restriction (CER), in which energy intake is restricted to below weight‐maintenance requirements over a continuous period of active weight loss effort. Such regimens typically consist of reducing daily energy intake by 15% to 60% [1]. Although CER is, on average, an effective weight loss strategy, there is significant interindividual variability reflecting individual challenges in adhering to the prescribed energy restriction [2, 3, 4]. Intermittent energy restriction (IER) regimens have been used as an alternative to CER and involve periods of energy restriction interrupted by periods of normal eating. IER has been proposed to increase dietary adherence due to its more flexible nature, which could lead to improved weight loss outcomes [5, 6, 7]. However, systematic reviews comparing IER with CER regimens have reported comparable weight loss [8, 9, 10, 11, 12, 13] and similar attrition rates [8, 12]. In these reviews, the IER regimens varied across studies, making it difficult to compare the effectiveness of different dietary regimens. However, the two most common and widely studied intermittent regimens, i.e., alternate day fasting and the 5:2 diet, lead to weight loss that is not different from that achieved with CER [1, 9, 10].

It is uncertain whether pausing a weight loss program for a defined longer period of time could improve weight loss. Such “planned pauses” in a weight loss program could permit greater flexibility, allow participants to regain motivation, and enhance subsequent dietary adherence, thereby improving weight loss outcomes. Because attrition could be an indicator of nonadherence to an intervention, lower attrition could also be indicative of a program that is easier to sustain, potentially resulting in better long‐term outcomes. On the other hand, extended pauses within a weight loss program may inadvertently lead to a decline in motivation and focus among participants, posing a risk of hindering overall weight loss progress. Therefore, identifying the optimal pause duration that balances both flexibility and sustained dietary adherence could be fundamental to the success of incorporating planned pauses within a weight loss regimen.

For the purposes of this review, we consider planned pauses to be distinct from intermittent dieting. Planned pauses were defined as intermittent intervals of no less than 1 week long in contrast to regimens within a 1‐week period (e.g., the 5:2 diet), which have been considered in previous reviews.

This systematic review and meta‐analysis of randomized‐controlled trials aimed to investigate the effect of planned pauses compared with CER, usual care, or minimal intervention on weight loss and attrition.

## METHODS

A protocol for this systematic review was registered prospectively on International Prospective Register of Systematic Reviews (PROSPERO) [14]. This review followed the Preferred Reporting Items for Systematic Reviews and Meta‐Analyses (PRISMA) reporting guidelines [15], with the checklist available in the online Supporting Information.

### Search strategy

MEDLINE, Embase, PsycINFO, Web of Science, and the Cochrane Central Register of Controlled Trials were searched from database inception to July 2023, using terms for the following concepts: “planned pauses”; “weight loss”; and “randomized‐controlled trials” (see online Supporting Information for all search strategies). References were also screened from systematic reviews identified through the search, and full‐text papers were requested from authors for those that we were unable to obtain. The searches were not restricted by country or language. The trial registries ClinicalTrials.gov and the World Health Organization (WHO) International Clinical Trials Registry were also searched. The reference lists of included studies were hand‐searched to identify any additional relevant studies.

Studies were included if they recruited adults (age ≥ 18 years) with a body mass index (BMI) ≥ 25 kg/m^2^ and had a randomized‐controlled design investigating a planned‐pause intervention during a weight loss program. The intervention needed to explicitly state that the aim was to reduce weight or maintain lost weight. A planned pause was defined as intermittent intervals of no less than 1 week long. To be considered a planned pause, the intervention must have included at least one pause accompanied by a return to broadly the same program. The initial energy restriction intervention must have been at least 1 week, and the pause interval could not be longer than the preceding intervention interval. Trials of interventions were included without restriction on the total length of the trial or the length of follow‐up of participants. Studies were required to report at least one measure of weight loss (e.g., body weight, BMI, percentage body weight) and to include a comparator arm of either CER (i.e., daily energy restriction), usual care, or a minimal intervention.

### Data collection

The Covidence (Cochrane) systematic review software was used for abstract and full‐text screening and data extraction [16]. Titles and abstracts were assessed for inclusion by two independent reviewers. Full‐text papers of the included studies were obtained and independently assessed for inclusion by two reviewers. Two reviewers independently conducted data extraction using a predetermined data extraction form, with data collected on the population, intervention, control groups, and outcomes. Any conflicts in screening or extraction were resolved by discussion, or, when needed, by referral to a third reviewer. Study authors were contacted when further detail and clarification was required.

The primary outcome was the mean weight change at the end of the intervention and at final follow‐up. When outcome data were missing, authors were contacted for further information. Weight change data was extracted as reported. When multiple methods were reported for participants lost to follow‐up, the most conservative estimate was extracted (i.e., intention‐to‐treat analysis). The secondary outcome was attrition at the end of the intervention and at final follow‐up; therefore, the numbers of participants at each time point were also extracted.

Risk of bias was assessed by two independent reviewers using the Cochrane risk of bias tool [17]. Risk of bias was assessed based on generation of the randomization sequence, concealment of allocation, blinding of outcome assessment, and attrition bias. It is not possible to blind participants or study personnel in behavioral intervention trials; therefore, this domain was omitted. The selective outcome reporting domain was also omitted because we were only interested in extracting measures of weight loss, as specified in the eligibility criteria. Studies were judged at high risk of attrition bias if less than 80% of participants were followed up at the end of the intervention, if less than 60% were followed up at the final follow‐up, or if the percentage followed up was different across trial arms (≥20% difference). Blinding of outcome assessment was judged as high risk if weight was self‐reported by the participant.

### Statistical analysis

Meta‐analyses were conducted in Review Manager version 5.2 (Cochrane) [18].The primary analysis aimed to examine the difference in weight change between the planned‐pause intervention and CER comparator groups at the end of the active intervention and at final follow‐up. Pooled results were calculated as mean differences in kilograms with 95% confidence intervals (CIs).

The secondary analysis aimed to compare the attrition rate between the planned‐pause intervention and CER comparator groups. For each study, the proportion of participants that dropped out in the intervention arm (*pI*) and the comparator arm (*pC*) were used to calculate a risk ratio (*pI/pC*) using the Mantel–Haenszel method. A risk ratio greater than one indicates greater attrition in the planned‐pause intervention arm compared with the comparator, and a risk ratio less than one indicates a lower attrition in the planned‐pause intervention arm compared with the comparator. Each risk ratio was weighted by the inverse variance and pooled to create a summary effect.

Subgroup analyses were used to test for differences in the effectiveness and the attrition rate of the planned‐pause intervention by calculating the mean difference in weight loss or risk ratio of attrition, and the *χ* ^2^ test was used for subgroup differences. Subgroups of studies were pooled by pause duration (short pauses of 1 week, medium pauses of 2 weeks, or long pauses of >4 weeks) and by the regimen used in the pause interval (maintain weight, “usual” diet, or energy restriction).

All meta‐analyses used a random‐effects model to account for variation in the intervention programs and populations being tested. Statistical heterogeneity was assessed using the I^2^ statistic [19]. When a study contributed more than one intervention arm to the analysis, the control group was divided equally between arms to avoid double counting in the pooled result. Planned sensitivity analyses excluded studies at high risk of bias in any domain. We also assessed the consistency and precision of the evidence. Consistency was based on the direction of the effect estimates. Analyses were judged as consistent if the direction of the effect was the same for each of the studies included in the analysis. Precision was based on the width of the CIs around the estimate of the effect. Visual assessment of funnel plots was used to determine the influence of publication bias.

## RESULTS

**FIGURE 1:**
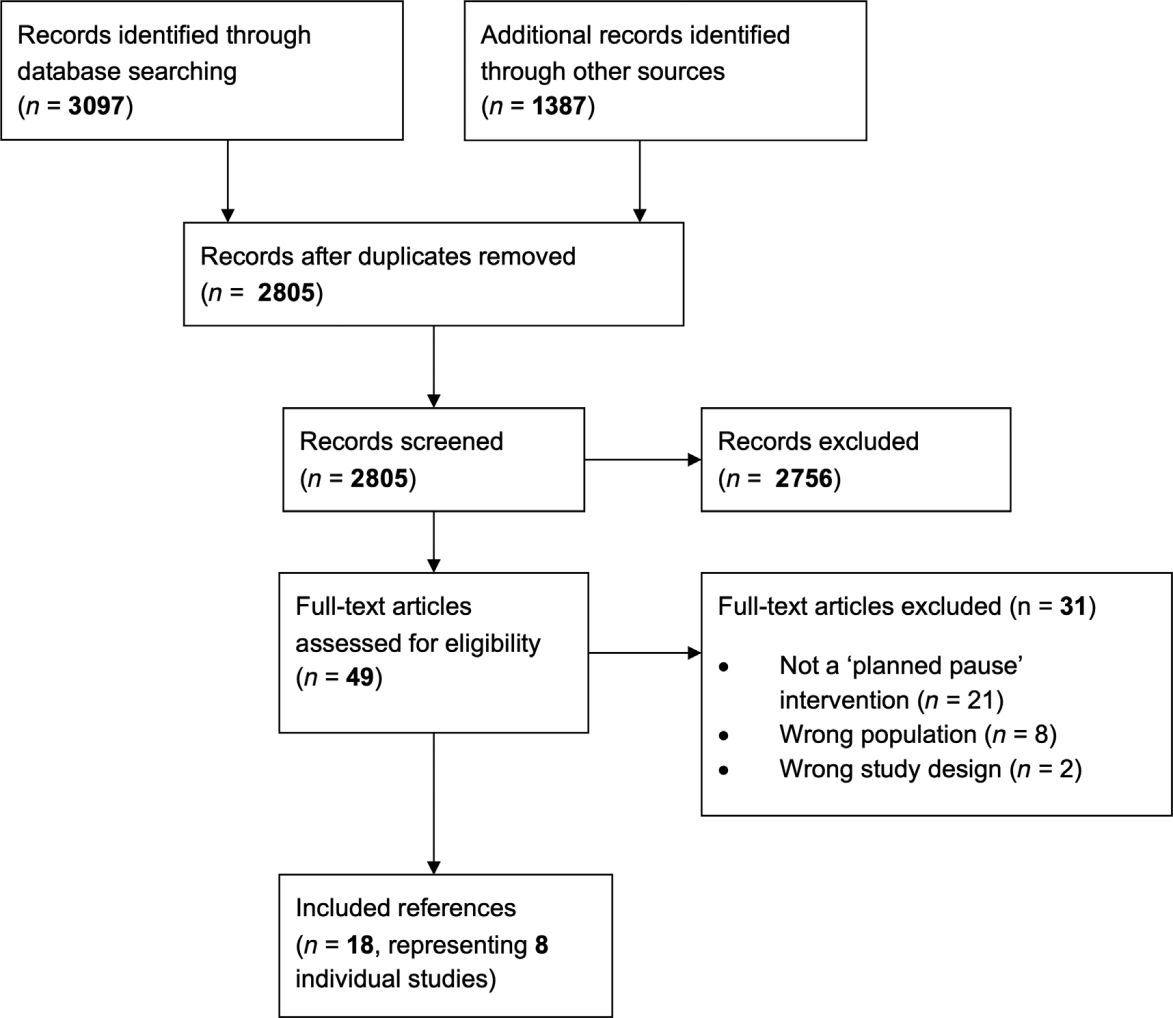
Preferred Reporting Items for Systematic Reviews and Meta‐Analyses (PRISMA) flow diagram of review process.

The literature search identified a total of 4484 records: 3097 identified from database searches, and 1387 from other sources. After title and abstract screening, 2756 references were excluded, and full‐text papers were retrieved for 49 references. A total of 31 references were excluded after full‐text screening. The main reason for exclusion was that the intervention did not meet the definition of a planned pause. Two studies that otherwise met our definition of a planned‐pause intervention were excluded due to being conducted in an ineligible population (i.e., BMI < 25 kg/m^2^) [20, 21]. Two studies that met all other inclusion criteria were excluded because the duration of the pause interval was longer than the preceding energy restriction interval [22, 23]. Ultimately, 18 references met the inclusion criteria, which represented eight individual studies (including 1 study abstract). The PRISMA flow chart is displayed in Figure 1.

### Characteristics of included studies

The eight included studies included 796 participants. The number of participants in each study ranged from 25 to 214, with an average of 100 participants per study. Four studies were conducted in Australia [24, 25, 26, 27], two studies in the United States [6, 28], one study in Canada [29], and one study in Turkey [30]. Four studies were conducted in people living with overweight or obesity [6, 24, 25, 30], three studies in people living with obesity [26, 27, 29], and one study in people who had overweight and type 2 diabetes [28].

Overall, 77% of participants were female, the mean age ranged from 36.0 to 60.5 years, and the mean BMI (reported in 7 studies) ranged from 30.5 to 37.9. Wing et al. had two intervention arms, which qualified as a planned‐pause intervention and thereby were compared with the CER control group separately [6]. For planned‐pause interventions, attrition rate ranged from 8% to 60% at the end of the active intervention and from 15% to 66% at the final follow‐up. For CER interventions, attrition rate ranged from 3% to 49% at the end of the active intervention and from 25% to 62% at the final follow‐up.

### Intervention and comparators

The median duration of the total intervention period in the nine included intervention arms was 26 weeks (range: 8–52 weeks). Six studies reported weight change at a further time point after the end of the active intervention, with a median final follow‐up of 52 weeks (range: 24–104 weeks) [6, 24, 25, 26, 28, 29]. Three interventions included short pause intervals of 1 week [24, 25, 30], three interventions included medium pauses intervals of 2 weeks [6, 26, 27], and three interventions included long pause intervals of 5 to 12 weeks [6, 28, 29]. Three interventions used maintenance energy requirements in the pause intervals, during which participants were advised to maintain their weight stable or were provided with 100% of their energy requirements [26, 27, 29]; five interventions guided participants to follow their “usual” diet during the pause interval [6, 24, 25, 30]; and, in one intervention, participants followed moderate energy restriction during the pause interval [28]. Two studies included food provision in both the intervention and control arms for the duration of the trial [26, 27].

In all eight included studies, the comparator arm included a CER intervention [6, 24, 25, 26, 27, 28, 29, 30]. None of the eligible studies included usual care or a minimal intervention as the comparator. The total intervention duration in the CER comparator arms ranged from 8 to 52 weeks. In all studies, the prescribed energy restriction during the restriction phase of the planned‐pause intervention was the same as the energy restriction in the CER arm. Further details of the planned‐pause and CER arms for each included study can be found in Table 1.

**TABLE 1:** Characteristics of included studies

Author, year (reference)	Country	Population	Control regimen	Planned‐pause regimen	Pause interval	Data collection time points	Attrition
Arguin, 2012 [29]	Canada	Postmenopausal women with obesity, *n* = 25 Mean age: 60.5 ± 6.0 y 100% female	*n* = 12, 15 wk of CER Dietitian prescribed energy restriction to reduce body weight by 1% of initial body weight per wk	*n* = 13, 3 × 5‐wk blocks of energy restriction alternating with 2 × 5‐wk blocks of weight maintenance Same energy restriction requirements as CER	Daily weighing Instructed to maintain stable body weight (±2 kg)	End of intervention: 20/30 wk Follow‐up: 12 mo	End of intervention: 8% planned pause; 17% CER Follow‐up: 15% planned pause; 25% CER
Byrne, 2018 [26]	Australia	Men with obesity, *n* = 51 Mean BMI: 34.5 ± 3.7 kg/m^2^ Mean age: 39.6 ± 8.2 y 0% female	*n* = 25, 16 wk of CER Weight‐maintenance energy requirements estimated by multiplying resting energy expenditure (measured via indirect calorimetry) with self‐reported physical activity level Food provided with 67% of weight‐maintenance energy requirements	*n* = 26, 8 × 2‐wk blocks of energy restriction alternating with 7 × 2‐wk blocks of energy balance Same energy restriction requirements as CER	Daily weighing Food provided with 100% of weight‐maintenance energy requirements	End of intervention: 16/30 wk Follow‐up: 48/62 wk	End of intervention: 27% planned pause; 12% CER Follow‐up: 42% planned pause; 48% CER
Byrne, 2020 [27]	Australia	Women with obesity, *n* = 52 Mean BMI: 35.0 ± 3.6 kg/m^2^ Mean age: 40.0 ± 7.0 y 100% female	*n* = 24, 12 wk of CER Weight‐maintenance energy requirements estimated by multiplying resting energy expenditure (measured via indirect calorimetry) with self‐reported physical activity level Food provided with 67% of weight‐maintenance energy requirements	*n* = 28, 6 × 2‐wk blocks of energy restriction alternating with 5 × 2‐wk blocks of energy balance Same energy restriction requirements as CER	Daily weighing Food provided with 100% of weight‐maintenance energy requirements	End of intervention: 12/22 wk Follow‐up: 6 mo	End of intervention: 43% planned pause; 29% CER Follow‐up: 64% planned pause; 42% CER
Erdem, 2022 [30]	Turkey	Men and women with overweight or obesity, *n* = 144 Mean BMI: 30.5 ± 2.6 kg/m^2^ Mean age: 36.0 ± 12.1 y 69% female	*n* = 72, 13 wk of CER Daily energy requirements calculated using the Schofield equation to calculate basal metabolic rate A Mediterranean diet that met 70% of energy requirements was prescribed	*n* = 72, 1‐wk Mediterranean energy restriction diet alternating with 1‐wk “normal” diet for 13 wk	Participants instructed to return to their usual diet	End of intervention: 13 wk	End of intervention: 24% planned pause; 3% CER
Headland, 2019 [24]	Australia	Men and women with overweight or obesity, *n* = 214 Mean BMI: 33.9 ± 5.3 kg/m^2^ Mean age: 50.3 ± 13.2 y 84% female	*n* = 104, 52 wk of CER Prescribed energy intake of 1000 kcal/d for women and 1200 kcal/d for men	*n* = 110, 1‐wk habitual diet alternating with 1‐wk energy restriction for 52 wk Same energy restriction requirements as CER	Participants instructed to return to their usual diet	End of intervention: 52 wk Follow‐up: 24 mo	End of intervention: 60% planned pause; 49% CER Follow‐up: 66% planned pause; 62% CER
Keogh, 2014 [25]	Australia	Women with overweight or obesity, *n* = 75 Mean BMI: 33.1 ± 5.8 kg/m^2^ Mean age: 60.1 ± 10.5 y 100% female	*n* = 36, 52 weeks of CER Research centre visits for first 8 wk Prescribed energy restriction of 1315 kcal/d	*n* = 39, 1‐wk habitual diet alternating with 1‐wk energy restriction for 52 wk Research centre visits for first 8 wk Same energy restriction requirements as CER	Participants instructed to return to their usual diet	End of intervention: 8 wk Follow‐up: 12 mo	8 wk: 36% planned pause; 44% CER Follow‐up: 51% planned pause; 53% CER
Wing, 1994 [28]	United States	Men and women with overweight and type 2 diabetes, *n* = 93 Mean BMI: 37.9 ± 6.3 kg/m^2^ Mean age: 51.8 ± 9.7 y 65% female	*n* = 48, 50 wk of moderate energy restriction Prescribed energy intake of 1000–1200 kcal/d	*n* = 45, severe energy restriction (400–500 kcal/d) for 2 × 12‐wk blocks alternating with 2 × 12‐wk blocks of moderate energy restriction (same energy requirements as CER)	Prescribed intake increased over a 4‐wk period until participants consumed 1000–1200 kcal/d (same as CER)	End of intervention: 50 wks Follow‐up: 24 mo	End of intervention: 16% planned pause; 15% CER Follow‐up: 20% planned pause; 23% CER
Wing and Jeffery, 2003 [6]	United States	Men and women with overweight or obesity, *n* = 142 Mean BMI: 33.1 ± 3.3 kg/m^2^ Mean age: 42.6 ± 9.3 y 85% female	*n* = 48, 14 wk of a behavioral weight loss program including CER Prescribed energy intake of 1000–1500 kcal/d depending on initial body weight	Long break, *n* = 47: 7 wk of energy restriction, 6‐wk break, 7 wk of energy restriction Short break, *n* = 47: 3 × 3‐wk blocks of energy restriction alternating with 3 × 2‐wk break, then 5 wk of energy restriction Same behavioral weight loss program and energy restriction requirements as CER	Participants instructed to stop all weight loss efforts and eat and exercise as before the program Participants instructed to not monitor their behaviors or weight	End of intervention: 20 wk Follow‐up: 11 mo	End of intervention: 6% long break; 23% short break; 21% CER Follow‐up: 32% long break; 30% short break; 36% CER

Abbreviation: CER, continuous energy restriction.

### Risk of bias

**FIGURE 2:**
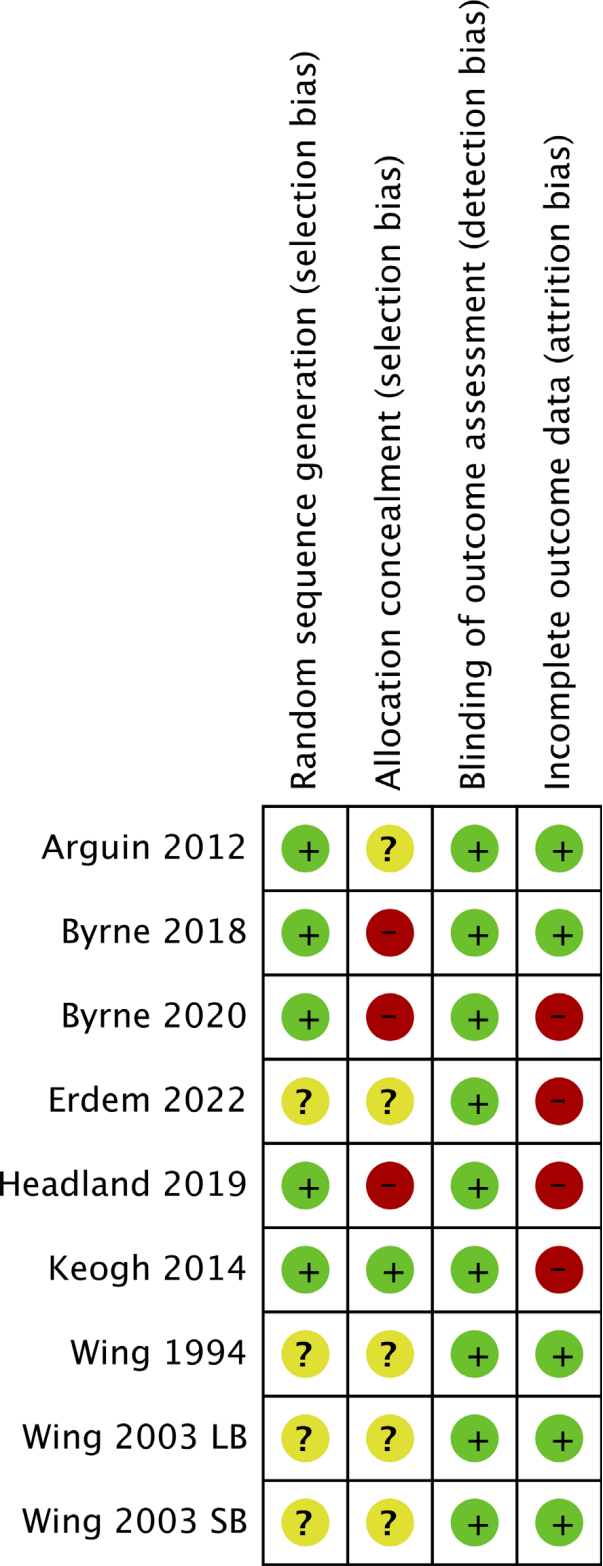
Risk of bias assessment for included studies. [Color figure can be viewed at wileyonlinelibrary.com]

Five interventions were judged to be at high risk of bias in at least one domain [24, 25, 26, 27, 30], with all others judged to be at unclear risk of bias in at least one domain. Four interventions were judged at unclear risk of random sequence generation bias owing to insufficient reporting [6, 28, 30]. Three interventions were judged as high risk of allocation concealment bias because allocation was not concealed [24, 26, 27], and five interventions were judged as unclear risk because they did not report whether allocation was concealed or not [6, 28, 29, 30]. Four studies were judged as high risk of attrition bias because <80% of participants were followed up at the end of the intervention, <60% were followed up at the final follow‐up, or the percentage followed up was different across trial arms (≥20% difference) [24, 25, 27, 30]. A summary of the risk of bias assessment for the included studies can be found in Figure 2. We were unable to formally test for publication bias due to the small number of included studies.

### Weight change

**FIGURE 3:**
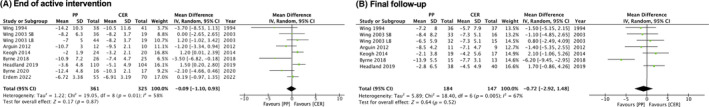
Forest plot showing mean difference in weight change (kilograms) between planned‐pause (PP) interventions versus continuous energy restriction (CER). (A) Weight change from baseline to the end of the active intervention. (B) Weight change from baseline to final follow‐up. [Color figure can be viewed at wileyonlinelibrary.com]

Eight studies (9 intervention arms) were included in the analysis examining weight change at the end of the active intervention (Figure 3A), and six studies (7 intervention arms) were included in the analysis at final follow‐up (Figure 3B). One study found significantly greater weight loss in the planned‐pause intervention compared with CER at the end of the active intervention and at final follow‐up [26]. However, pooled results revealed no difference in weight change between the planned‐pause intervention group and the CER control group at the end of the intervention or at final follow‐up. At the end of the active intervention, mean weight change among participants in the planned‐pause intervention group (*n* = 361) was −7.09 kg compared with the participants in the CER group (*n* = 325), who had a weight change of −7.0 kg (mean difference: −0.09 kg, 95% CI: −1.10 to 0.93). At the final follow‐up, mean weight change among participants in the planned‐pause intervention group (*n* = 184) was −6.91 kg compared with the participants in the CER group (*n* = 147), who had a weight change of −6.19 kg (mean difference: −0.72 kg, 95% CI: −2.92 to 1.48). In planned sensitivity analyses, removing studies with high risk of bias in at least one category did not meaningfully affect the results.

Overall, the evidence was mostly consistent but imprecise for weight change at both time points. Statistical heterogeneity was moderately high, with an *I* ^2^ of 58% and a Tau^2^ of 1.22 for the end of intervention and an *I* ^2^ of 67% and a Tau^2^ of 5.89 for the final follow‐up. This was not unexpected due to the variability in the planned‐pause regimen among studies, and this was explored further in subgroup analyses.

#### Pause interval duration

**FIGURE 4:**
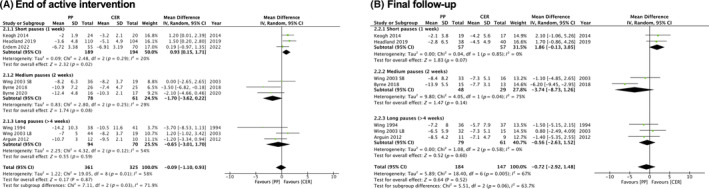
Forest plot showing mean difference in weight change (kilograms) between planned‐pause (PP) interventions versus continuous energy restriction (CER) by pause interval duration. (A) Weight change from baseline to the end of the active intervention. (B) Weight change from baseline to final follow‐up. [Color figure can be viewed at wileyonlinelibrary.com]

Interventions with similar pause interval duration were grouped for analysis. There were significant between‐group differences overall at the end of the active intervention (Figure 4A), but not at final follow‐up (Figure 4B). There was consistent and precise evidence from three studies that short pause intervals of 1 week led to less weight loss in the planned‐pause intervention compared with the control group at the end of the active intervention (mean difference: 0.93 kg, 95% CI: 0.15 to 1.71, *I* ^2^ = 20%). Two of these studies also reported weight change at a further follow‐up time in which no difference in weight change was detected between the planned‐pause intervention or the control group (mean difference: 1.86 kg, 95% CI: −0.13 to 3.85, *I* ^2^ = 0%). Based on consistent but imprecise evidence, there was no difference in weight change between the planned‐pause intervention and the control group for medium pause intervals of 2 weeks or longer pause intervals of >4 weeks at either the end of the active intervention or at final follow‐up (*n* = 3 studies for each interval). Significant subgroup differences at the end of the active intervention were not maintained at final follow‐up.

#### Dietary regimen in pause interval

**FIGURE 5:**
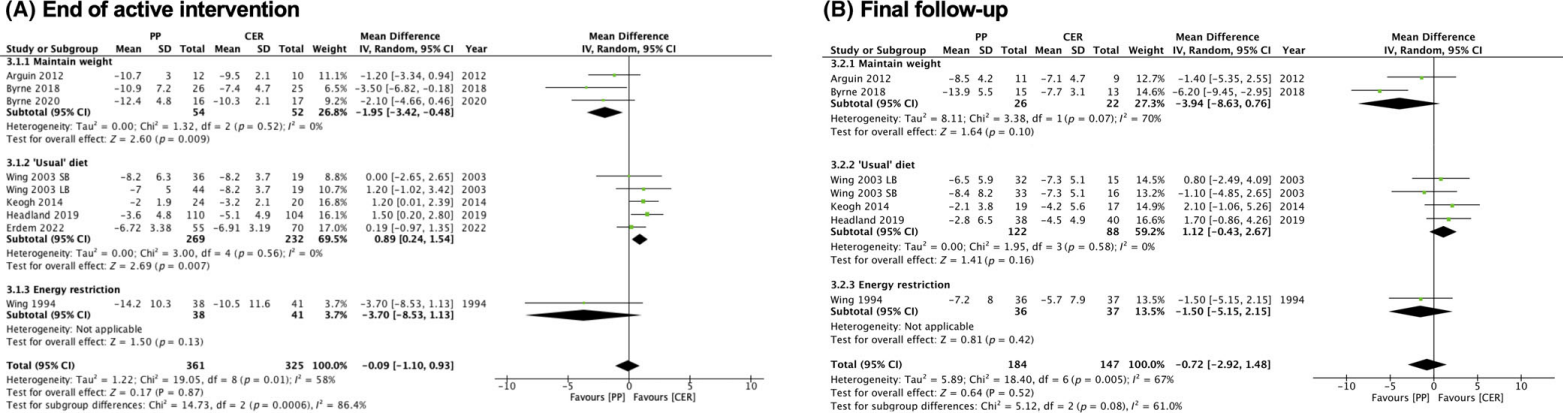
Forest plot showing mean difference in weight change (kilograms) between planned‐pause (PP) interventions versus continuous energy restriction (CER) by dietary regimen used in the pause interval. (A) Weight change from baseline to the end of the active intervention. (B) Weight change from baseline to final follow‐up. [Color figure can be viewed at wileyonlinelibrary.com]

Different dietary regimens used in the pause interval were grouped for analysis. There were significant between‐group differences overall at the end of the active intervention (Figure 5A), but not at final follow‐up (Figure 5B). There was consistent but imprecise evidence that intervention arms that advised patients to maintain their weight stable during the pause interval led to greater weight loss in the planned‐pause intervention compared with CER (mean difference: −1.95 kg, 95% CI: −3.42 to −0.48, *I* ^2^ = 0%, *n* = 3 studies). Two of these studies also reported weight change at a further follow‐up time in which no difference in weight change was detected between the planned‐pause intervention or the control group (mean difference: −3.94 kg, 95% CI: −8.63 to 0.76, *I* ^2^ = 70%). Based on consistent and precise evidence, arms that instructed participants to follow their usual diet during the pause interval led to less weight loss in the planned‐pause intervention compared with the control group (mean difference: 0.89 kg, 95% CI: 0.24 to 1.54, *I* ^2^ = 0%, *n* = 5 studies). Four of these studies also reported weight change at a further follow‐up time in which no difference in weight change was detected between the planned‐pause intervention or the control group (mean difference: 1.12 kg, 95% CI: −0.43 to 2.67, *I* ^2^ = 0%). Although there were significant differences between subgroups at the end of the active intervention, these were not maintained at the final follow‐up.

No difference in weight change between the planned‐pause intervention and the CER control group was found at either the end of the active intervention or at final follow‐up for the one study arm in which participants followed a moderate energy restriction during the pause interval.

### Attrition

**FIGURE 6:**
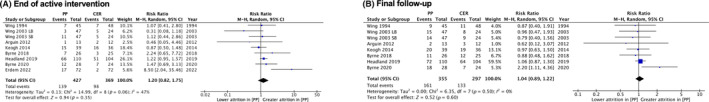
Forest plot showing mean risk ratio of attrition for planned‐pause (PP) interventions versus continuous energy restriction (CER). (A) Risk of attrition from baseline to the end of the active intervention. (B) Risk of attrition from baseline to final follow‐up. A risk ratio greater than one indicates greater attrition in the PP arm compared with CER, and a risk ratio less than one indicates lower attrition in the PP arm compared with CER. [Color figure can be viewed at wileyonlinelibrary.com]

Nine intervention arms reported the proportion of participants who dropped out at the end of the active intervention and eight intervention arms at a further follow‐up. Greater attrition in the planned‐pause group compared with the CER group was found in one study at the end of the active intervention [30] and in one study at the final follow‐up [27]. However, overall, the pooled risk ratio showed no difference in attrition rate in the planned‐pause intervention arms versus the CER arms at the end of the active intervention (risk ratio: 1.20, 95% CI: 0.82 to 1.75, *I* ^2^ = 47%; Figure 6A) or at final follow‐up (risk ratio: 1.04, 95% CI: 0.89 to 1.22, *I* ^2^ = 0%; Figure 6B). Overall, the evidence was mostly consistent but imprecise for attrition at both time points. In subgroup analyses, there were also no significant between‐group differences in attrition rate when studies were categorized by pause duration or the dietary regimen used in the pause interval.

## DISCUSSION

This systematic review and meta‐analysis illustrated that weight loss programs incorporating planned pauses led to clinically significant weight loss but no different from CER interventions, with no evidence that planned pauses reduced attrition. There was some evidence that, when participants were guided to maintain their weight stable in the pause intervals, weight loss was improved. Short pauses (<1 week) were less effective than both continuous dieting and longer pauses during the active intervention, but not at final follow‐up.

Most previous systematic reviews and meta‐analyses have reported comparable weight losses for IER compared with CER regimens [8, 9, 10, 11, 12, 13]. The results presented here are in agreement with previous conclusions, despite differences in the definition of the IER intervention. Here, we defined a planned pause as intermittent intervals of no less than 1 week long, during which the pause interval could not be longer than the preceding intervention interval. This choice was derived from the aim of studying interventions that simulated, as much as possible, a planned pause in a weight loss program. Although this definition was chosen to be specifically different than intermittent dieting within a 1‐week period (e.g., the 5:2 diet), the overall conclusions are largely similar [1, 9, 10]. Another systematic review investigated similar intermittent intervals of ≥7 days but operationalized this differently, i.e., with no limitations on the energy restriction period relative to the pause period, and also found comparable weight loss with CER [31]. Absolute mean weight losses in both the planned‐pause and CER groups were >5 kg at the end of the active intervention and at final follow‐up, suggesting that both interventions could be effective strategies for weight loss.

Given the scope of the review, there was a large degree of variability in the planned‐pause regimen in the included studies, reflected by high statistical heterogeneity. To investigate this, subgroup analyses were conducted to investigate potential explanations for this variation in weight losses. Pauses of 1 week resulted in less weight loss compared with CER, which could possibly suggest that a 1‐week interval is insufficient to provide a sufficient break to recharge motivation for weight loss. At the end of the active intervention, interventions in which participants were guided to maintain their weight in the pause interval resulted in greater weight loss, whereas interventions that guided participants to follow their “usual” diet in the pause interval resulted in lower levels of weight loss compared with CER. These findings are in accordance with a previous review, which suggested that the key differentiator among studies that reported greater weight losses in IER interventions compared with CER was the nature of the weight‐maintenance phase [32].

Subgroup differences were small and only apparent at the end of the active intervention and not maintained over longer periods of time. One study reported a much larger mean weight loss of −13.9 kg in the planned‐pause group compared with −7.7 kg in the CER group at their final follow‐up time point [26], but this study supplied all food to participants, and it is uncertain whether the same results could be achieved in routine practice without food provision. Overall, there was little evidence that planned pauses provide participants with a more sustainable strategy for weight control beyond the end of the intervention period compared with traditional dietary approaches.

We hypothesized that planned pauses could be a strategy to reduce attrition and achieve longer‐term engagement, thereby improving weight loss. However, attrition was comparable between planned‐pause interventions and CER at each time point. These results are in agreement with other reviews comparing CER and IER interventions [8, 12], as well as other weight loss programs that show attrition rates of 30% to 60% [8, 33, 34]. Given that attrition could be an indicator of nonadherence to an intervention, these results provide no clear evidence that planned pauses are easier to adhere to than CER. However, there was also no clear evidence that they were inferior, meaning that they could be an effective option for some people.

In this controlled research setting with a median follow‐up time of 52 weeks, there was no evidence that planned pauses were more effective than CER. However, in a more pragmatic, self‐directed context with longer follow‐up, planned pauses may permit greater flexibility, maximizing dietary adherence and thereby allowing individuals to maintain a period of active weight management for a longer period of time. This could be important in managing obesity as a chronic relapsing condition; however, further research is required to draw definitive conclusions.

This review has several strengths. The scope of this review differs from previous reviews that have investigated the effect of shorter periods of IER, thereby providing novel insights into the effectiveness of implementing planned pauses into a weight loss program. To minimize bias and confounding, this review included only randomized‐controlled trials and had no restrictions on year, language, or intervention duration. Only trials that compared planned pauses with a control arm were included, which allowed the estimation of effect sizes through the inclusion of meta‐analyses. We were also able to conduct exploratory subgroup analyses to assess whether the planned‐pause regimen impacted the effectiveness of the intervention.

However, this review also has some limitations. Because a specific definition of planned pauses was used, only nine intervention arms met the inclusion criteria, each with moderate sample sizes and subsequent small numbers in each subgroup, meaning one group could not be pooled for meta‐analysis. Additional studies identified during the systematic review search included pause intervals that were longer than the preceding intervention interval; these were not considered a planned pause by our definition and were thereby excluded. Although we attempted to group similar planned‐pause dietary regimens in the subgroup analyses, pooled interventions were still highly variable in prescribed energy restriction, intervention duration, and timing of the intermittent periods, reflected by the heterogeneity within some subgroups. Inadequate reporting prevented clear assessment of the quality of included studies, particularly random sequence generation and allocation concealment methods, meaning the possibility of selection bias cannot be ruled out. Studies in the review also had considerable attrition, and only two out of the nine intervention arms reported intention‐to‐treat analyses at the end of the active intervention, and one reported it at the final follow‐up. Owing to the relatively small number of studies, we were unable to formally test for publication bias. Finally, the majority of participants included in this review were female, limiting the generalizability of the findings to male individuals.

In conclusion, for people living with overweight or obesity, planned pauses were found to be consistently no more effective than CER to achieve weight loss, with no evidence that planned pauses reduced attrition. Longer pauses and advice to maintain weight stable during the pause may improve the effectiveness of the planned pause.

## FUNDING INFORMATION

Gina M. Wren is funded by the Medical Research Council (MRC) (grant number: MR/R015708/1). Dimitrios A. Koutoukidis is funded by a National Institute for Health Research (NIHR) Advanced Fellowship (grant number: NIHR302549). Jadine Scragg and Susan A. Jebb are funded by the NIHR Oxford Biomedical Research Centre (grant number: IS‐BRC‐1215‐20008). Elena Tsompanaki is funded by the Novo Nordisk UK Research Foundation with a Fellowship in Clinical Diabetes. Alice Hobson is funded by NIHR Applied Research Collaborations Oxford. The funders had no role in the design and conduct of the study; collection, management, analysis, and interpretation of the data; preparation, review, or approval of the manuscript; and decision to submit the manuscript for publication. The views expressed are those of the authors and not necessarily those of the MRC, the NHS, the NIHR, the Department of Health and Social Care, or the Novo Nordisk UK Research Foundation.

## CONFLICT OF INTEREST STATEMENT

Second Nature is the commercial partner on Gina M. Wren's MRC industrial collaborative awards in science and engineering (iCASE) studentship but had no involvement in this review. Dimitrios A. Koutoukidis and Susan A. Jebb report being investigators in two investigator‐led, publicly funded (NIHR) trials in which the weight loss intervention was donated by Nestlé Health Science and Oviva Therepeutics to the University of Oxford outside the submitted work. Susan A. Jebb and Jadine Scragg report being investigators in a trial in which Second Nature is delivering the weight loss intervention. Elena Tsompanaki reports being an investigator in a trial in which hemoglobin A1c testing kits were donated by Medichecks to the University of Oxford outside the submitted work. Elena Tsompanaki and Dimitrios A. Koutoukidis report being investigators in an investigator‐led weight loss trial in which meal‐replacement products were purchased from Nestlé Health Science at a discounted rate. None of these associations led to payments to these authors personally. Jadine Scragg was a member of a Nestlé Health Sciences advisory board and was an invited speaker at a Nestlé Health Sciences webinar, for which a personal honorarium was received.

## Supporting information


**Data S1.** Supporting information.
