# Supplementary material for: Effect of planned pauses versus continuous energy restriction on weight loss and attrition: a systematic review
Source: Obesity (Silver Spring). 2024 Jan 21;32(3):454–65. doi: 10.1002/oby.23976 (PMC11423418; doi:10.1002/oby.23976)
Supplement: Supplementary file 1 — Data S1. Supporting information. [file OBY-32-454-s001.docx]

**Supplementary Materials**

**Effect of planned pauses versus continuous energy restriction on weight loss and attrition: a systematic review**

Gina M Wren ^1*^, Dimitrios A Koutoukidis ^1^, Jadine Scragg ^1,2^, Elena Tsompanaki ^1^, Alice Hobson ^1^, Susan A Jebb ^1,2^

^1^Nuffield Department of Primary Care Health Sciences, University of Oxford, Radcliffe Observatory Quarter, Woodstock Road, Oxford, OX2 6GG

^2^ NIHR Oxford Biomedical Research Centre, Oxford University Hospitals NHS Foundation Trust, Oxford, Oxfordshire, UK

***Corresponding Author**: Gina M Wren, Nuffield Department of Primary Care Health Sciences

[gina.wren@phc.ox.ac.uk](mailto:gina.wren@phc.ox.ac.uk)

**Search Strategies**

|  | **Medline** |
| --- | --- |
|  |  |
| 1 | Weight Reduction Programs/ and (intermittent or break? or pause? or interrupt? or disrupt?).mp. |
| 2 | (caloric restriction/ or diet, reducing/) and (intermittent or break? or pause? or interrupt? or disrupt?).mp. |
| 3 | (((calor* or energy or diet) adj3 (restrict* or reduc*)) and (intermittent or break? or pause? or interrupt? or disrupt?)).mp. |
| 4 | (dieting and (intermittent or break? or pause? or interrupt? or disrupt?)).mp. |
| 5 | (intermittent adj5 (fast* or diet* or restrict* or food depriv* or calori* or low-calorie*)).mp. |
| 6 | (intermittent energy adj5 (fast* or diet* or restrict* or food depriv*)).mp. |
| 7 | (periodic adj5 (fast* or diet* or restrict* or food depriv*)).mp. |
| 8 | ((pause? or break? or interrupt? or disrupt?) adj5 (weight adj3 (chang* or loss or maintain* or maintenance or control or management))).mp. |
| 9 | ((pause? or break? or interrupt? or disrupt?) adj5 ((weight or obes* or overweight) adj10 (intervention? or program*))).mp. |
| 10 | ((pause? or break? or interrupt? or disrupt?) adj5 ((lifestyle or life style or behav* or diet* or nutrition* or health* eating) adj10 (intervention? or program*))).mp. |
| 11 | 1 or 2 or 3 or 4 or 5 or 6 or 7 or 8 or 9 or 10 |
| 12 | body weight changes/ or weight loss/ |
| 13 | body mass index/ or waist-hip ratio/ or Waist Circumference/ or exp Body Composition/ |
| 14 | (weight adj3 (chang* or loss or lose or losing or maintain* or maintenance or decreas* or reduc* or improv*)).ti,ab,kw. |
| 15 | (body weight or body fat or fat mass or body composition or body mass index or bmi or "waist hip ratio" or "waist to hip ratio" or waist circumference).ti,ab,kw. |
| 16 | 12 or 13 or 14 or 15 |
| 17 | 11 and 16 |
| 18 | randomized controlled trial.pt. |
| 19 | controlled clinical trial.pt. |
| 20 | randomized.ab. |
| 21 | placebo.ab. |
| 22 | drug therapy.fs. |
| 23 | randomly.ab. |
| 24 | trial.ab. |
| 25 | groups.ab. |
| 26 | 18 or 19 or 20 or 21 or 22 or 23 or 24 or 25 |
| 27 | exp animals/ not humans.sh. |
| 28 | 26 not 27 |
| 29 | 17 and 28 |

|  | **Embase** |
| --- | --- |
|  |  |
| 1 | weight loss program/ AND (intermittent OR break? OR pause? OR interrupt? OR disrupt?).mp. |
| 2 | (caloric restriction/ or diet restriction/ or low calorie diet/) AND (intermittent OR break? OR pause? OR interrupt? OR disrupt?).mp. |
| 3 | (((calor* OR energy OR diet) ADJ3 (restrict* OR reduc*)) AND (intermittent OR break? OR pause? OR interrupt? OR disrupt?)).mp. |
| 4 | (dieting AND (intermittent OR break? OR pause? OR interrupt? OR disrupt?)).mp. |
| 5 | (intermittent ADJ5 (fast* OR diet* OR restrict* OR "food depriv*" OR calori* OR low-calorie*)).mp. |
| 6 | ("intermittent energy" ADJ5 (fast* OR diet* OR restrict* OR "food depriv*")).mp. |
| 7 | (periodic ADJ5 (fast* OR diet* OR restrict* OR "food depriv*")).mp. |
| 8 | ((pause? OR break? OR interrupt? OR disrupt?) ADJ5 (weight ADJ3 (chang* OR loss OR maintain* OR maintenance OR control OR management))).mp. |
| 9 | ((pause? OR break? OR interrupt? OR disrupt?) ADJ5 ((weight OR obes* OR overweight) ADJ10 (intervention? OR program*))).mp. |
| 10 | ((pause? OR break? OR interrupt? OR disrupt?) ADJ5 ((lifestyle OR "life style" OR behav* OR diet* OR nutrition* OR "health* eating") ADJ10 (intervention? OR program*))).mp. |
| 11 | 1 or 2 or 3 or 4 or 5 or 6 or 7 or 8 or 9 or 10 |
| 12 | body weight change/ OR body weight loss/ |
| 13 | abdominal circumference/ or body mass/ or body weight/ or waist circumference/ or waist hip ratio/ or waist to height ratio/ or exp body composition/ or fat mass/ or fat free mass/ |
| 14 | (weight ADJ3 (chang* OR loss OR lose OR losing OR maintain* OR maintenance OR decreas* OR reduc* OR improv*)).tw,kw. |
| 15 | (body weight OR body fat OR fat mass OR body composition OR body mass index OR bmi OR "waist hip ratio" OR "waist to hip ratio" OR "waist circumference").tw,kw. |
| 16 | 12 or 13 or 14 or 15 |
| 17 | 11 and 16 |
| 18 | randomized controlled trial/ or controlled clinical trial/ |
| 19 | (random* or placebo).ti,ab. |
| 20 | randomization/ or intermethod comparison/ or human experiment/ or double blind procedure/ |
| 21 | (compare or compared or comparison).ti. or ((evaluated or evaluate or evaluating or assessed or assess) and (compare or compared or comparing or comparison)).ab. |
| 22 | (open adj label).ti,ab. |
| 23 | ((double or single or doubly or singly) adj (blind or blinded or blindly)).ti,ab. |
| 24 | parallel group*1.ti,ab. |
| 25 | (crossover or cross over).ti,ab. |
| 26 | ((assign$ or match or matched or allocation) adj5 (alternate or group$1 or intervention$1 or patient$1 or subject$1 or participant$1)).ti,ab. |
| 27 | (assigned or allocated).ti,ab. |
| 28 | (controlled adj7 (study or design or trial)).ti,ab. or trial.ti. |
| 29 | (volunteer or volunteers).ti,ab. |
| 30 | 18 or 19 or 20 or 21 or 22 or 23 or 24 or 25 or 26 or 27 or 28 or 29 |
| 31 | (rat or rats or mouse or mice or swine or porcine or murine or sheep or lambs or pigs or piglets or rabbit or rabbits or cat or cats or dog or dogs or cattle or bovine or monkey or monkeys or trout or marmoset$1).ti. and animal experiment/ |
| 32 | Animal experiment/ not (human experiment/ or human/) |
| 33 | 31 or 32 |
| 34 | 30 not 33 |
| 35 | 17 and 34 |

|  | **PsycINFO** |
| --- | --- |
|  |  |
| 1 | weight control/ AND (intermittent.mp. OR break?.mp. OR pause?.mp. OR interrupt?.mp. OR disrupt?.mp.) |
| 2 | (((calor*.mp. OR energy.mp. OR diet.mp.) ADJ3 (restrict*.mp. OR reduc*.mp.)) AND (intermittent.mp. OR break?.mp. OR pause?.mp. OR interrupt?.mp. OR disrupt?.mp.)) |
| 3 | (dieting.mp. AND (intermittent.mp. OR break?.mp. OR pause?.mp. OR interrupt?.mp. OR disrupt?.mp.)) |
| 4 | (intermittent.mp. ADJ5 (fast*.mp. OR diet*.mp. OR restrict*.mp. OR "food depriv*".mp. OR calori*.mp. OR low-calorie*.mp.)) |
| 5 | ("intermittent energy".mp. ADJ5 (fast*.mp. OR diet*.mp. OR restrict*.mp. OR "food depriv*".mp.)) |
| 6 | (periodic.mp. ADJ5 (fast*.mp. OR diet*.mp. OR restrict*.mp. OR "food depriv*".mp.)) |
| 7 | ((pause?.mp. OR break?.mp. OR interrupt?.mp. OR disrupt?.mp.) ADJ5 (weight.mp. ADJ3 (chang*.mp. OR loss.mp. OR maintain*.mp. OR maintenance.mp. OR control.mp. OR management.mp.))) |
| 8 | ((pause?.mp. OR break?.mp. OR interrupt?.mp. OR disrupt?.mp.) ADJ5 ((weight.mp. OR obes*.mp. OR overweight.mp.) ADJ10 (intervention?.mp. OR program*.mp.))) |
| 9 | ((pause?.mp. OR break?.mp. OR interrupt?.mp. OR disrupt?.mp.) ADJ5 ((lifestyle.mp. OR "life style".mp. OR behav*.mp. OR diet*.mp. OR nutrition*.mp. OR "health* eating".mp.) ADJ10 (intervention?.mp. OR program*.mp.))) |
| 10 | 1 or 2 or 3 or 4 or 5 or 6 or 7 or 8 or 9 |
| 11 | body weight/ OR weight loss/ |
| 12 | body mass index/ OR body fat/ |
| 13 | (weight adj3 (chang* or loss or lose or losing or maintain* or maintenance or decreas* or reduc* or improv*)).ti,ab. |
| 14 | ("body weight" OR "body fat" OR "fat mass" OR "body composition" OR "body mass index" OR bmi OR "waist hip ratio" OR "waist to hip ratio" OR "waist circumference").ti,ab. |
| 15 | 11 or 12 or 13 or 14 |
| 16 | 10 and 15 |
| 17 | random*.ti,ab,hw,id. |
| 18 | trial*.ti,ab,hw,id. |
| 19 | controlled stud*.ti,ab,hw,id. |
| 20 | placebo*.ti,ab,hw,id. |
| 21 | ((singl* or doubl* or trebl* or tripl*) and (blind* or mask*)).ti,ab,hw,id. |
| 22 | (cross over or crossover or factorial* or latin square).ti,ab,hw,id. |
| 23 | (assign* or allocat* or volunteer*).ti,ab,hw,id. |
| 24 | treatment effectiveness evaluation/ |
| 25 | mental health program evaluation/ |
| 26 | exp experimental design/ |
| 27 | (clinical trial or treatment outcome).md. |
| 28 | 17 or 18 or 19 or 20 or 21 or 22 or 23 or 24 or 25 or 26 or 27 |
| 29 | (animals or  animal  or  mice  or  mus  or  mouse  or  murine  or  woodmouse  or  rats  or  rat  or  murinae  or  muridae  or  cottonrat  or  cottonrats  or  hamster  or  hamsters  or  cricetinae  or  rodentia  or  rodent  or  rodents  or  pigs  or  pig  or  swine  or  swines  or  piglets  or  piglet  or  boar  or  boars  or  sus  scrofa  or  ferrets  or  ferret  or  polecat  or  polecats  or  mustela  putorius  or  guinea  pigs  or  guinea  pig  or  cavia  or  callithrix  or  marmoset  or  marmosets  or  cebuella  or  hapale  or  octodon  or  chinchilla  or  chinchillas  or  gerbillinae  or  gerbil  or  gerbils  or  jird  or  jirds  or  merione  or  meriones  or  rabbits  or  rabbit  or  hares  or  hare  or  diptera  or  flies  or  fly  or  dipteral  or  drosphila  or  drosophilidae  or  cats  or  cat  or  carus  or  felis  or  nematoda  or  nematode  or  nematoda  or  nematode  or  nematodes  or  sipunculida  or  dogs  or  dog  or  canine  or  canines  or  canis  or  sheep  or  sheeps  or  mouflon  or  mouflons  or  ovis  or  goats  or  goat  or  capra  or  capras  or  rupicapra  or  chamois  or  haplorhini  or  monkey  or  monkeys  or  anthropoidea  or  anthropoids  or  saguinus  or  tamarin  or  tamarins  or  leontopithecus  or  hominidae  or  ape  or  apes  or  pan  or  paniscus  or  pan  paniscus  or  bonobo  or  bonobos  or  troglodytes  or  pan  troglodytes  or  gibbon  or  gibbons  or  siamang  or  siamangs  or  nomascus  or  symphalangus  or  chimpanzee  or  chimpanzees  or  prosimians  or  bush  baby  or  prosimian  or  bush  babies  or  galagos  or  galago  or  pongidae  or  gorilla  or  gorillas  or  pongo  or  pygmaeus  or  pongo  pygmaeus  or  orangutans  or  pygmaeus  or  lemur  or  lemurs  or  lemuridae  or  horse  or  horses  or  pongo  or  equus  or  cow  or  calf  or  bull  or  chicken  or  chickens  or  gallus  or  quail  or  bird  or  birds  or  quails  or  poultry  or  poultries  or  fowl  or  fowls  or  reptile  or  reptilia  or  reptiles  or  snakes  or  snake  or  lizard  or  lizards  or  alligator  or  alligators  or  crocodile  or  crocodiles  or  turtle  or  turtles  or  amphibian  or  amphibians  or  amphibia  or  frog  or  frogs  or  bombina  or  salientia  or  toad  or  toads  or  epidalea  calamita  or  salamander  or  salamanders  or  eel  or  eels  or  fish  or  fishes  or  pisces  or  catfish  or  catfishes  or  siluriformes  or  arius  or  heteropneustes  or  sheatfish  or  perch  or  perches  or  percidae  or  perca  or  trout  or  trouts  or  char  or  chars  or  salvelinus  or  fathead  minnow  or  minnow  or  cyprinidae  or  carps  or  carp  or  zebrafish  or  zebrafishes  or  goldfish  or  goldfishes  or  guppy  or  guppies  or  chub  or  chubs  or  tinca  or  barbels  or  barbus  or  pimephales  or  promelas  or  poecilia  reticulata  or  mullet  or  mullets  or  seahorse  or  seahorses  or  mugil  curema  or  atlantic  cod  or  shark  or  sharks  or  catshark  or  anguilla  or  salmonid  or  salmonids  or  whitefish  or  whitefishes  or  salmon  or  salmons  or  sole  or  solea  or  sea  lamprey  or  lamprey  or  lampreys  or  pumpkinseed  or  sunfish  or  sunfishes  or  tilapia  or  tilapias  or  turbot  or  turbots  or  flatfish  or  flatfishes  or  sciuridae  or  squirrel  or  squirrels  or  chipmunk  or  chipmunks  or  suslik  or  susliks  or  vole  or  voles  or  lemming  or  lemmings  or  muskrat  or  muskrats  or  lemmus  or  otter  or  otters  or  marten  or  martens  or  martes  or  weasel  or  badger  or  badgers  or  ermine  or  mink  or  minks  or  sable  or  sables  or  gulo  or  gulos  or  wolverine  or  wolverines  or  minks  or  mustela  or  llama  or  llamas  or  alpaca  or  alpacas  or  camelid  or  camelids  or  guanaco  or  guanacos  or  chiroptera  or  chiropteras  or  bat  or  bats  or  fox  or  foxes  or  iguana  or  iguanas  or  xenopus  laevis  or  parakeet  or  parakeets  or  parrot  or  parrots  or  donkey  or  donkeys  or  mule  or  mules  or  zebra  or  zebras  or  shrew  or  shrews  or  bison  or  bisons  or  buffalo  or  buffaloes  or  deer  or  deers  or  bear  or  bears  or  panda  or  pandas  or  wild  hog  or  wild  boar  or  fitchew  or  fitch  or  beaver  or  beavers  or  jerboa  or  jerboas  or  capybara  or  capybaras).ti,ab. |
| 30 | 28 not 29 |
| 31 | \| 16 and 30 \| \| --- \| |

|  | **Cochrane** |
| --- | --- |
|  |  |
| #1 | [mh "weight reduction program"] AND (intermittent or break* or pause* or interrupt* or disrupt*):ti,ab,kw |
| #2 | [mh "caloric restriction"] AND (intermittent or break* or pause* or interrupt* or disrupt*):ti,ab,kw |
| #3 | ((((calor* or energy or diet) NEAR/3 (restrict* or reduc*)) AND (intermittent or break* or pause* or interrupt* or disrupt*))):ti,ab,kw |
| #4 | (dieting and (intermittent or break* or pause* or interrupt* or disrupt*)):ti,ab,kw |
| #5 | ((intermittent NEAR/5 (fast* or diet* or restrict* or food depriv* or calori* or low-calorie*))):ti,ab,kw |
| #6 | (("intermittent energy" NEAR/5 (fast* or diet* or restrict* or food depriv*))):ti,ab,kw |
| #7 | ((periodic NEAR/5 (fast* or diet* or restrict* or food depriv*))):ti,ab,kw |
| #8 | (((intermittent or break* or pause* or interrupt* or disrupt*) NEAR/5 (weight NEAR/3 (chang* or loss or maintain* or maintenance or control or management)))):ti,ab,kw |
| #9 | (((intermittent or break* or pause* or interrupt* or disrupt*) NEAR/5 ((weight or obes* or overweight) NEAR/10 (intervention* or program*)))):ti,ab,kw |
| #10 | (((intermittent or break* or pause* or interrupt* or disrupt*) NEAR/5 ((lifestyle or life style or behav* or diet* or nutrition* or healthful eating or healthy eating) NEAR/10 (intervention* or program*)))):ti,ab,kw |
| #11 | #1 or #2 or #3 or #4 or #5 or #6 or #7 or #8 or #9 or #10 |
| #12 | [mh "body weight changes"] OR [mh "weight loss"] OR [mh "waist-hip ratio"] OR [mh "Waist Circumference"] OR [mh "Body Composition"] |
| #13 | ((weight NEAR/3 (chang* or loss or lose or losing or maintain* or maintenance or decreas* or reduc* or improv*))):ti,ab,kw |
| #14 | (("body weight" or "body fat" or "fat mass" or "body composition" or "body mass index" or bmi or "waist hip ratio" or "waist to hip ratio" or "waist circumference")):ti,ab,kw |
| #15 | #12 or #13 or #14 |
| #16 | #11 and #15 |

|  | **Web of Science** |
| --- | --- |
| 1 | TS=(((calor* OR energy OR diet) NEAR/3 (restrict* OR reduc*)) AND (intermittent OR break$ OR pause$ OR interrupt$ OR disrupt$)) |
| 2 | TS=(dieting AND (intermittent OR break$ OR pause$ OR interrupt$ OR disrupt$)) |
| 3 | TS=(intermittent NEAR/5 (fast* OR diet* OR restrict* OR "food depriv*" OR calori* OR low-calorie*)) |
| 4 | TS=("intermittent energy" NEAR/5 (fast* OR diet* OR restrict* OR "food depriv*")) |
| 5 | TS=(periodic NEAR/5 (fast* OR diet* OR restrict* OR "food depriv*")) |
| 6 | TS=((pause$ OR break$ OR interrupt$ OR disrupt$) NEAR/5 (weight NEAR/3 (chang* OR loss OR maintain* OR maintenance OR control OR management))) |
| 7 | TS=((pause$ OR break$ OR interrupt$ OR disrupt$) NEAR/5 ((weight OR obes* OR overweight) NEAR/10 (intervention$ OR program*))) |
| 8 | TS=((pause$ OR break$ OR interrupt$ OR disrupt$) NEAR/5 ((lifestyle OR "life style" OR behav* OR diet* OR nutrition* OR "health* eating") NEAR/10 (intervention$ OR program*))) |
| 9 | 1 or 2 or 3 or 4 or 5 or 6 or 7 or 8 |
| 10 | TS = (weight NEAR/3 (chang* OR loss OR lose OR losing OR maintain* OR maintenance OR decreas* OR reduc* OR improv*)) |
| 11 | TS=("body weight" OR "body fat" OR "fat mass" OR "body composition" OR "body mass index" OR bmi OR "waist hip ratio" OR "waist to hip ratio" OR "waist circumference") |
| 12 | 10 or 11 |
| 13 | 9 and 12 |
| 14 | TS=(random* or blind* or allocat* or assign* or trial* or placebo* or crossover* or cross-over*) |
| 15 | TS=(animals or  animal  or  mice  or  mus  or  mouse  or  murine  or  woodmouse  or  rats  or  rat  or  murinae  or  muridae  or  cottonrat  or  cottonrats  or  hamster  or  hamsters  or  cricetinae  or  rodentia  or  rodent  or  rodents  or  pigs  or  pig  or  swine  or  swines  or  piglets  or  piglet  or  boar  or  boars  or  sus  scrofa  or  ferrets  or  ferret  or  polecat  or  polecats  or  mustela  putorius  or  guinea  pigs  or  guinea  pig  or  cavia  or  callithrix  or  marmoset  or  marmosets  or  cebuella  or  hapale  or  octodon  or  chinchilla  or  chinchillas  or  gerbillinae  or  gerbil  or  gerbils  or  jird  or  jirds  or  merione  or  meriones  or  rabbits  or  rabbit  or  hares  or  hare  or  diptera  or  flies  or  fly  or  dipteral  or  drosphila  or  drosophilidae  or  cats  or  cat  or  carus  or  felis  or  nematoda  or  nematode  or  nematoda  or  nematode  or  nematodes  or  sipunculida  or  dogs  or  dog  or  canine  or  canines  or  canis  or  sheep  or  sheeps  or  mouflon  or  mouflons  or  ovis  or  goats  or  goat  or  capra  or  capras  or  rupicapra  or  chamois  or  haplorhini  or  monkey  or  monkeys  or  anthropoidea  or  anthropoids  or  saguinus  or  tamarin  or  tamarins  or  leontopithecus  or  hominidae  or  ape  or  apes  or  pan  or  paniscus  or  pan  paniscus  or  bonobo  or  bonobos  or  troglodytes  or  pan  troglodytes  or  gibbon  or  gibbons  or  siamang  or  siamangs  or  nomascus  or  symphalangus  or  chimpanzee  or  chimpanzees  or  prosimians  or  bush  baby  or  prosimian  or  bush  babies  or  galagos  or  galago  or  pongidae  or  gorilla  or  gorillas  or  pongo  or  pygmaeus  or  pongo  pygmaeus  or  orangutans  or  pygmaeus  or  lemur  or  lemurs  or  lemuridae  or  horse  or  horses  or  pongo  or  equus  or  cow  or  calf  or  bull  or  chicken  or  chickens  or  gallus  or  quail  or  bird  or  birds  or  quails  or  poultry  or  poultries  or  fowl  or  fowls  or  reptile  or  reptilia  or  reptiles  or  snakes  or  snake  or  lizard  or  lizards  or  alligator  or  alligators  or  crocodile  or  crocodiles  or  turtle  or  turtles  or  amphibian  or  amphibians  or  amphibia  or  frog  or  frogs  or  bombina  or  salientia  or  toad  or  toads  or  epidalea  calamita  or  salamander  or  salamanders  or  eel  or  eels  or  fish  or  fishes  or  pisces  or  catfish  or  catfishes  or  siluriformes  or  arius  or  heteropneustes  or  sheatfish  or  perch  or  perches  or  percidae  or  perca  or  trout  or  trouts  or  char  or  chars  or  salvelinus  or  fathead  minnow  or  minnow  or  cyprinidae  or  carps  or  carp  or  zebrafish  or  zebrafishes  or  goldfish  or  goldfishes  or  guppy  or  guppies  or  chub  or  chubs  or  tinca  or  barbels  or  barbus  or  pimephales  or  promelas  or  poecilia  reticulata  or  mullet  or  mullets  or  seahorse  or  seahorses  or  mugil  curema  or  atlantic  cod  or  shark  or  sharks  or  catshark  or  anguilla  or  salmonid  or  salmonids  or  whitefish  or  whitefishes  or  salmon  or  salmons  or  sole  or  solea  or  sea  lamprey  or  lamprey  or  lampreys  or  pumpkinseed  or  sunfish  or  sunfishes  or  tilapia  or  tilapias  or  turbot  or  turbots  or  flatfish  or  flatfishes  or  sciuridae  or  squirrel  or  squirrels  or  chipmunk  or  chipmunks  or  suslik  or  susliks  or  vole  or  voles  or  lemming  or  lemmings  or  muskrat  or  muskrats  or  lemmus  or  otter  or  otters  or  marten  or  martens  or  martes  or  weasel  or  badger  or  badgers  or  ermine  or  mink  or  minks  or  sable  or  sables  or  gulo  or  gulos  or  wolverine  or  wolverines  or  minks  or  mustela  or  llama  or  llamas  or  alpaca  or  alpacas  or  camelid  or  camelids  or  guanaco  or  guanacos  or  chiroptera  or  chiropteras  or  bat  or  bats  or  fox  or  foxes  or  iguana  or  iguanas  or  xenopus  laevis  or  parakeet  or  parakeets  or  parrot  or  parrots  or  donkey  or  donkeys  or  mule  or  mules  or  zebra  or  zebras  or  shrew  or  shrews  or  bison  or  bisons  or  buffalo  or  buffaloes  or  deer  or  deers  or  bear  or  bears  or  panda  or  pandas  or  wild  hog  or  wild  boar  or  fitchew  or  fitch  or  beaver  or  beavers  or  jerboa  or  jerboas  or  capybara  or  capybaras) |
| 16 | 14 NOT 15 |
| 17 | 16 AND 13 |

**Trial Registers**

| **WHOICTRP** |
| --- |
| (intermittent OR break OR breaks OR pause OR paused OR interrupt OR interrupted OR interruption OR disrupt OR disrupted OR disruption) AND (Weight loss" OR "lose weight" OR "losing weight" OR "Weight change" OR "maintain weight" OR "weight maintenance" OR "weight reduction" OR "weight control") |
| (intermittent OR break OR breaks OR pause OR paused OR interrupt OR interrupted OR interruption OR disrupt OR disrupted OR disruption) AND (bmi OR "body mass index" OR "body fat" OR "body composition" OR "waist hip ratio" OR "waist to hip ratio" OR "waist circumference") |

| **Clinicaltrials.gov** |
| --- |
| Other Terms=intermittent OR break OR breaks OR pause OR paused OR interrupt OR interrupted OR interruption OR disrupt OR disrupted OR disruption AND Outcome="Weight loss" OR "lose weight" OR "losing weight" OR "Weight change" OR "maintain weight" OR "weight maintenance" OR "weight reduction" OR "weight control" |
| Interventions=intermittent OR break OR breaks OR pause OR paused OR interrupt OR interrupted OR interruption OR disrupt OR disrupted OR disruption AND Outcome="Weight loss" OR "lose weight" OR "losing weight" OR "Weight change" OR "maintain weight" OR "weight maintenance" OR "weight reduction" OR "weight control" |
| Interventions=intermittent OR break OR breaks OR pause OR paused OR interrupt OR interrupted OR interruption OR disrupt OR disrupted OR disruption AND Outcome=bmi OR "body mass index" OR "body fat" OR "body composition" OR "waist hip ratio" OR "waist to hip ratio" OR "waist circumference" |
| Other Terms=intermittent OR break OR breaks OR pause OR paused OR interrupt OR interrupted OR interruption OR disrupt OR disrupted OR disruption AND Outcome=bmi OR "body mass index" OR "body fat" OR "body composition" OR "waist hip ratio" OR "waist to hip ratio" OR "waist circumference" |

**PRISMA Checklist**

| **Section and Topic** | **Item #** | **Checklist item** | **Location where item is reported** |
| --- | --- | --- | --- |
| **TITLE** | | |  |
| Title | 1 | Identify the report as a systematic review. | Title Page |
| **ABSTRACT** | | |  |
| Abstract | 2 | See the PRISMA 2020 for Abstracts checklist. | Page 1 |
| **INTRODUCTION** | | |  |
| Rationale | 3 | Describe the rationale for the review in the context of existing knowledge. | Page 2-3 |
| Objectives | 4 | Provide an explicit statement of the objective(s) or question(s) the review addresses. | Page 3 |
| **METHODS** | | |  |
| Eligibility criteria | 5 | Specify the inclusion and exclusion criteria for the review and how studies were grouped for the syntheses. | Page 4 |
| Information sources | 6 | Specify all databases, registers, websites, organisations, reference lists and other sources searched or consulted to identify studies. Specify the date when each source was last searched or consulted. | Page 3-4 |
| Search strategy | 7 | Present the full search strategies for all databases, registers and websites, including any filters and limits used. | Supplementary material |
| Selection process | 8 | Specify the methods used to decide whether a study met the inclusion criteria of the review, including how many reviewers screened each record and each report retrieved, whether they worked independently, and if applicable, details of automation tools used in the process. | Page 4 |
| Data collection process | 9 | Specify the methods used to collect data from reports, including how many reviewers collected data from each report, whether they worked independently, any processes for obtaining or confirming data from study investigators, and if applicable, details of automation tools used in the process. | Page 5 |
| Data items | 10a | List and define all outcomes for which data were sought. Specify whether all results that were compatible with each outcome domain in each study were sought (e.g. for all measures, time points, analyses), and if not, the methods used to decide which results to collect. | Page 5 |
|  | 10b | List and define all other variables for which data were sought (e.g. participant and intervention characteristics, funding sources). Describe any assumptions made about any missing or unclear information. | Page 5 |
| Study risk of bias assessment | 11 | Specify the methods used to assess risk of bias in the included studies, including details of the tool(s) used, how many reviewers assessed each study and whether they worked independently, and if applicable, details of automation tools used in the process. | Page 5-6 |
| Effect measures | 12 | Specify for each outcome the effect measure(s) (e.g. risk ratio, mean difference) used in the synthesis or presentation of results. | Page 6 |
| Synthesis methods | 13a | Describe the processes used to decide which studies were eligible for each synthesis (e.g. tabulating the study intervention characteristics and comparing against the planned groups for each synthesis (item #5)). | Page 4-5 |
|  | 13b | Describe any methods required to prepare the data for presentation or synthesis, such as handling of missing summary statistics, or data conversions. | Page 4-5 |
|  | 13c | Describe any methods used to tabulate or visually display results of individual studies and syntheses. | Page 4-5 |
|  | 13d | Describe any methods used to synthesize results and provide a rationale for the choice(s). If meta-analysis was performed, describe the model(s), method(s) to identify the presence and extent of statistical heterogeneity, and software package(s) used. | Page 6-7 |
|  | 13e | Describe any methods used to explore possible causes of heterogeneity among study results (e.g. subgroup analysis, meta-regression). | Page 6-7 |
|  | 13f | Describe any sensitivity analyses conducted to assess robustness of the synthesized results. | Page 7 |
| Reporting bias assessment | 14 | Describe any methods used to assess risk of bias due to missing results in a synthesis (arising from reporting biases). | Page 3 |
| Certainty assessment | 15 | Describe any methods used to assess certainty (or confidence) in the body of evidence for an outcome. | Page 4 |
| **RESULTS** | | |  |
| Study selection | 16a | Describe the results of the search and selection process, from the number of records identified in the search to the number of studies included in the review, ideally using a flow diagram. | Page 7 and Fig 1 |
|  | 16b | Cite studies that might appear to meet the inclusion criteria, but which were excluded, and explain why they were excluded. | Page 7 |
| Study characteristics | 17 | Cite each included study and present its characteristics. | Page 8 and Table 1 |
| Risk of bias in studies | 18 | Present assessments of risk of bias for each included study. | Page 10 |
| Results of individual studies | 19 | For all outcomes, present, for each study: (a) summary statistics for each group (where appropriate) and (b) an effect estimate and its precision (e.g. confidence/credible interval), ideally using structured tables or plots. | Figures 3-6 |
| Results of syntheses | 20a | For each synthesis, briefly summarise the characteristics and risk of bias among contributing studies. | Page 8-10 |
|  | 20b | Present results of all statistical syntheses conducted. If meta-analysis was done, present for each the summary estimate and its precision (e.g. confidence/credible interval) and measures of statistical heterogeneity. If comparing groups, describe the direction of the effect. | Page 10-13 |
|  | 20c | Present results of all investigations of possible causes of heterogeneity among study results. | Page 10-13 |
|  | 20d | Present results of all sensitivity analyses conducted to assess the robustness of the synthesized results. | Page 11 |
| Reporting biases | 21 | Present assessments of risk of bias due to missing results (arising from reporting biases) for each synthesis assessed. | Page 10 & Fig 2 |
| Certainty of evidence | 22 | Present assessments of certainty (or confidence) in the body of evidence for each outcome assessed. | Page 10-13 |
| **DISCUSSION** | | |  |
| Discussion | 23a | Provide a general interpretation of the results in the context of other evidence. | Page 13-14 |
|  | 23b | Discuss any limitations of the evidence included in the review. | Page 16-17 |
|  | 23c | Discuss any limitations of the review processes used. | Page 16-17 |
|  | 23d | Discuss implications of the results for practice, policy, and future research. | Page 16 |
| **OTHER INFORMATION** | | |  |
| Registration and protocol | 24a | Provide registration information for the review, including register name and registration number, or state that the review was not registered. | Page 3 |
|  | 24b | Indicate where the review protocol can be accessed, or state that a protocol was not prepared. | Page 3 |
|  | 24c | Describe and explain any amendments to information provided at registration or in the protocol. | n/a |
| Support | 25 | Describe sources of financial or non-financial support for the review, and the role of the funders or sponsors in the review. | Title page |
| Competing interests | 26 | Declare any competing interests of review authors. | Title page |
| Availability of data, code and other materials | 27 | Report which of the following are publicly available and where they can be found: template data collection forms; data extracted from included studies; data used for all analyses; analytic code; any other materials used in the review. | Supplementary material |

*From:*  Page MJ, McKenzie JE, Bossuyt PM, Boutron I, Hoffmann TC, Mulrow CD, et al. The PRISMA 2020 statement: an updated guideline for reporting systematic reviews. BMJ 2021;372:n71. doi: 10.1136/bmj.n71

For more information, visit: <http://www.prisma-statement.org/>
